# A delta-radiomics model for preoperative prediction of invasive lung adenocarcinomas manifesting as radiological part-solid nodules

**DOI:** 10.3389/fonc.2022.927974

**Published:** 2022-11-16

**Authors:** Wufei Chen, Ruizhi Wang, Zhuangxuan Ma, Yanqing Hua, Dingbiao Mao, Hao Wu, Yuling Yang, Cheng Li, Ming Li

**Affiliations:** Department of Radiology, Huadong Hospital, Fudan University, Shanghai, China

**Keywords:** PSNs, lung adenocarcinoma, CECT, delta-radiomics, LASSO-logistic

## Abstract

**Purpose:**

This study aims to explore the value of the delta-radiomics (DelRADx) model in predicting the invasiveness of lung adenocarcinoma manifesting as radiological part-solid nodules (PSNs).

**Methods:**

A total of 299 PSNs histopathologically confirmed as lung adenocarcinoma (training set, *n* = 209; validation set, *n* = 90) in our hospital were retrospectively analyzed from January 2017 to December 2021. All patients underwent diagnostic noncontrast-enhanced CT (NCECT) and contrast-enhanced CT (CECT) before surgery. After image preprocessing and ROI segmentation, 740 radiomic features were extracted from NCECT and CECT, respectively, resulting in 740 DelRADx. A DelRADx model was constructed using the least absolute shrinkage and selection operator logistic (LASSO-logistic) algorithm based on the training cohort. The conventional radiomics model based on NCECT was also constructed following the same process for comparison purposes. The prediction performance was assessed using area under the ROC curve (AUC). To provide an easy-to-use tool, a radiomics-based integrated nomogram was constructed and evaluated by integrated discrimination increment (IDI), calibration curves, decision curve analysis (DCA), and clinical impact plot.

**Results:**

The DelRADx signature, which consisted of nine robust selected features, showed significant differences between the AIS/MIA group and IAC group (*p* < 0.05) in both training and validation sets. The DelRADx signature showed a significantly higher AUC (0.902) compared to the conventional radiomics model based on NCECT (AUC = 0.856) in the validation set. The IDI was significant at 0.0769 for the integrated nomogram compared with the DelRADx signature. The calibration curve of the integrated nomogram demonstrated favorable agreement both in the training set and validation set with a mean absolute error of 0.001 and 0.019, respectively. Decision curve analysis and clinical impact plot indicated that if the threshold probability was within 90%, the integrated nomogram showed a high clinical application value.

**Conclusion:**

The DelRADx method has the potential to assist doctors in predicting the invasiveness for patients with PSNs. The integrated nomogram incorporating the DelRADx signature with the radiographic features could facilitate the performance and serve as an alternative way for determining management.

## Introduction

With the popularity of low-dose CT for lung cancer screening in recent years, a large number of early-stage lung adenocarcinomas radiographically manifesting as partial solid nodules (PSNs) have been screened out (1). Given the distinct surgical management strategy and prognosis for disease-free survival (DFS) and overall survival (OS), differentiation of the invasive adenocarcinoma (IAC) from the adenocarcinoma *in situ* (AIS) or minimally invasive adenocarcinoma (MIA) for this distinct subtype has been confirmed to be of great clinical significance (2). The malignant potential of PSNs in CT images has not been clarified; however, the evaluation of invasive qualities was primarily imaging- or clinic-based at the present stage, which is nonquantifiable and subjective (3, 4). Both radiologists and surgeons desire a precise and practical way to help guide clinical decision-making for the invasiveness of PSNs when faced with a such diagnostic quandary.

Histological evidence has revealed a tight relationship between the solid component of PSNs and the invasive component of adenocarcinomas (5, 6). The extra blood supply information of solid components may benefit from contrast-enhanced CT (CECT), which also brings out tumor heterogeneity (7, 8). Furthermore, a new high-throughput radiomics analysis method called delta-radiomics (DelRADx), which deals with a string of eigenvalue changes within different modes, has reportedly been linked to the efficacy or prognosis for malignancies such as colorectal, liver, or lung cancer (9–11). However, the use of DelRADx for the prediction of invasive adenocarcinomas manifested as PSNs is rarely reported. The purpose of this study is to develop a DelRADx model based on CECT and noncontrast-enhanced CT (NCECT) data in order to provide patients with PSNs for better decision support.

## Materials and methods

### Patients

Patients who underwent surgical resections for lung cancer at our hospital between January 2017 and December 2021 were reviewed retrospectively. The inclusion criteria were as follows: (1) pathologically confirmed as lung adenocarcinoma; (2) radiographically represented as PSNs (diameter, ≤3 cm) in axial CT at lung window setting; (3) NCECT and CECT images were obtained at one examination; and (4) thin-slice CT images (1–1.25 mm) could be obtained. The exclusion criteria were as follows: (1) nodule diameter less than 6 mm, due to the inherent calibration error of solid components within the threshold; (2) poor CT quality, such as severe motion artifacts; and (3) failure to extract the radiomic features for unknown reasons. For the multiple PSNs in one patient, only the lesion with the pathologically conclusive result was included.

Consequently, 299 cases with 299 PSNs who met the principles were enrolled in the cohort for the current analysis (detailed in **Figure S1**). According to the histopathological examination, 159 cases were diagnosed with AIS/MIA, while, 140 cases were diagnosed with IAC. The cases were randomly divided into training and validation sets in a ratio of 7:3. This study design was approved by the institutional research ethics board of our institution, and the informed consent requirement was waived for the retrospective research with anonymous data.

### Image acquisition and radiographic feature assessment

NCECT examinations were conducted in the supine position with the arms up after deep inspiration. The CT data were acquired from one of the two scanners: Somatom Definition flash (Siemens Medical Solutions, Germany) and GE Discovery CT750 HD scanner (GE Healthcare, USA). The scanning parameters are detailed in **Supplementary Table S1**. Subsequently, the CECT was performed at 35 to 60s after injecting a dose of 80–100 ml nonionic IV contrast material (350 mg/ml, Omnipaque, GE Healthcare) mixed with isotonic saline into the ulnar vein using a high-pressure syringe at a rate of 3.0–4.0 ml/s.

The clinical and radiographic features were reviewed by two radiologists (H.W. and D.B.M.) with more than 10 years of experience in chest CT interpretation in a blinded fashion. We used the electronic caliper in our picture archiving and communication system to measure the maximum diameter of PSNs (nodule_max) and the maximum diameter of the corresponding solid component (solid_max). The consolidation-to-tumor ratio (CTR) was subsequently determined by dividing the solid_max by the nodule_max. Any discrepancies in describing the radiographic features were settled by consensus reading.

### Nodule segmentation

The regions of interest (ROI) were manually contoured slicewise by one radiologist (Z.X.M.) with 5 years of experience to achieve three-dimensional segmentation using the open-source medical image processing and navigation software 3D slicer (version 4.8.0, Brigham and Women’s Hospital). Another radiologist (Y.L.Y.) with 6 years of experience segmented a random set of 20 nodules independently to assess the interobserver robustness of radiomic features. All ROI were exported in Nrrd (desensitization format) for the following analysis.

### Image preprocessing and radiomics feature extraction

All images were isotropically resampled with 1.0 mm at X/Y/Z-spacing using linear interpolation to standardize. The Gaussian filter was used to reduce the noise influence of the voxel on radiomic features. Feature extraction was performed with pyRadiomics (https://doi.org/10.1158/0008-5472.CAN-17-0339). The DelRADx was defined as the change of radiomic features from NCECT to CECT: DelRADx = Feature_CECT_ − Feature_NCECT_.

### Feature selection and modeling

The training set was used for feature selection. Robust features with an interclass correlation coefficient (ICC) of >0.8 were chosen for further analysis. The discriminative ability was first evaluated using the Mann–Whitney *U* test. Features with statistical significance were taken for the following analysis. The correlation matrix with the pair-wise Spearman correlation analysis was built to eliminate the redundant features (correlation coefficient >0.90). The least absolute shrinkage and selection operator (LASSO) logistic regression algorithm with fivefold cross-validation was used to develop a predictive DelRADx signature by linear summing the core DelRADx multiplied by their coefficient. Another conventional radiomics model based on the NCECT features was constructed by using the same flow process to evaluate the optimal predictive model.

### Statistical analysis

The feature selection, modeling, and statistical analysis were performed with R software (version 3.6.2; http://www.Rproject.org) or SPSS 21.0 (IBM, Chicago, IL, USA). The “irr” package was used for ICC. LASSO analysis was performed with the “glmnet” package. The nomogram and calibration curve were plotted based on the “rms” package. Decision curve analysis (DCA) and clinical impact plot were done with the “rmda” package. Multivariate binary logistic regression analysis was performed with an input parameter strategy. The model performance between the two radiomics models was evaluated by the ROC analysis. The significant difference was evaluated using the DeLong method. Integrated discrimination increment (IDI) was used to analyze the improvement of the integrated nomogram. A *p*-value of < 0.05 was considered statistically significant. All *p*-values were two sided in this study.

## Results

### Patients’ general characteristics

The general characteristics of 299 patients in the training and validation sets are summarized in **Table 1**. In the univariate analysis, there were no significant differences in gender, age, location, margin, air bronchogram, and vacuole sign (*p >*0.05). Statistically significant differences could be observed in the nodule_max, solid_max, and CTR both in the training and validation sets (*p* < 0.05). Consequently, these three quantitative parameters were chosen to establish a radiographic model. According to the univariate logistic regression analysis, only solid_max could independently predict the invasiveness of PSNs (**Table S2**).

**Table 1:** Demographic and radiographic characteristics of enrolled patients.

Variable	Training set			Validation set		
	AIS/MIA	IAC	p	AIS/MIA	IAC	p
Gender						
Male	32 (43.2)	42 (56.8)	0.134	14 (46.7)	16 (53.3)	0.068
Female	73 (54.1)	62 (45.9)		40 (66.7)	20 (33.3)	
Age	52.38 ± 12.20	54.93 ± 11.10	0.115	57.50 ± 10.14	58.06 ± 9.67	0.796
Solid_max	3.56 ± 0.72	5.07 ± 1.30	**0.0001** ^*^	4.28 ± 0.98	5.79 ± 1.33	**0.0001** ^*^
Nodule_max	9.30 ± 2.45	10.55 ± 2.64	**0.001**	10.67 ± 2.62	12.31 ± 3.16	**0.009**
CTR	0.39 ± 0.08	0.49 ± 0.10	**0.0001** ^*^	0.41 ± 0.07	0.49 ± 0.12	**0.0001** ^*^
Location						
RUL	41 (52.6)	37 (47.4)	0.632	16 (69.6)	7 (30.4)	0.103*
RML	7 (46.7)	8 (53.3)		1 (12.5)	7 (87.5)	
RLL	24 (58.5)	17 (41.5)		8 (47.1)	9 (52.9)	
LUL	23 (43.4)	30 (56.6)		23 (69.7)	10 (30.3)	
LLL	10 (45.5)	12 (54.5)		6 (66.7)	3 (33.3)	
Margin						
Blurred	40 (44.0)	51 (56.0)	0.111	20 (60.6)	13 (39.4)	0.929
Clear	65 (55.1)	53 (44.9)		34 (59.6)	23 (40.4)	
Air bronchogram						
Present	9 (75.0)	3 (25.0)	0.077	7 (77.8)	2 (22.2)	0.254*
Absent	96 (48.7)	101 (51.3)		47 (58.0)	34 (42.0)	
Vacuole sign						
Present	9 (50.0)	9 (50.0)	0.983	2 (66.7)	1 (33.3)	0.811*
Absent	96 (50.3)	95 (49.7)		52 (59.8)	35 (40.2)	

Data in parentheses are percentages. Fisher’s exact test results are marked by asterisks. Significant results (p < 0.05) are bolded.

^*^Mann–Whitney U test for the abnormal distribution.

The AUC of the radiographic model based on the binary logistic regression analysis was 0.890 (95% CI, 0.843–0.938) in the training set and 0.835 (95% CI, 0.745–0.926) in the validation set. The associated criterion of solid_max was 4.53 mm.

### Feature selection and DelRADx signature building

**Figure 1:**
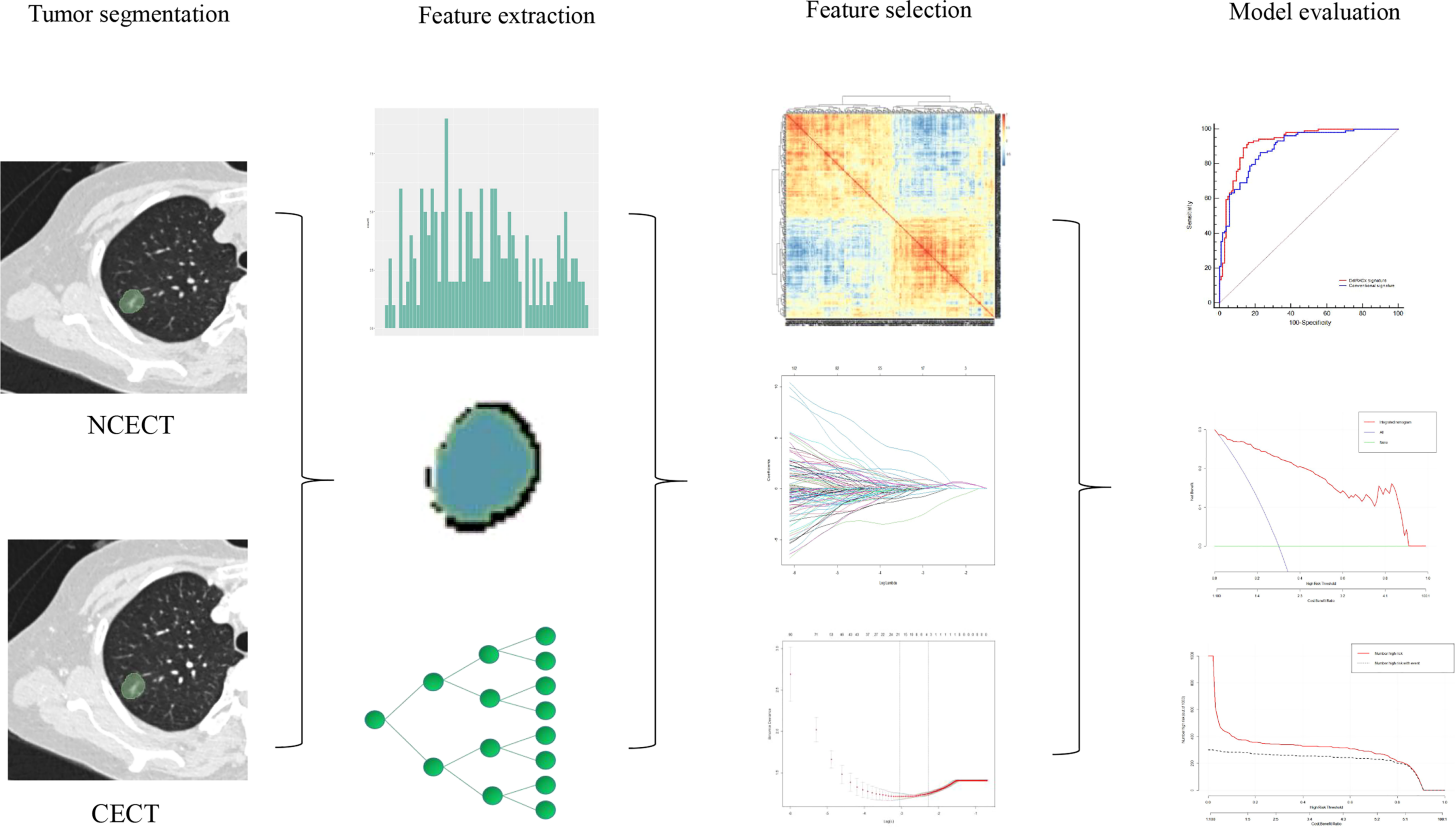
The schematic depiction of radiomics modeling.

A total of 740 radiomic features were extracted from the ROI of NCECT and CECT, respectively, resulting in 740 DelRADx. The schematic depiction of radiomics modeling was illustrated in **Figure 1**. After the process of ICC, ANOVA/MW, Spearman rank correlation test, and LASSO logistic regression analysis (detailed in **Figure S2**), nine robust DelRADx were ultimately selected. Based on the coefficients, the DelRADx signature was calculated for each patient. The signature formulas are provided in **Figure S3**.

Both the DelRADx and conventional radiomics signatures were significantly different between IAC and the AIS/MIA in the training set (0.78 ± 0.23 *vs*. 0.22 ± 0.25, 0.74 ± 0.24 *vs*. 0.26 ± 0.26) and the validation set (0.64 ± 1.25 *vs*. −1.99 ± 1.96, 0.44 ± 0.82 *vs*. −0.84 ± 0.81) (*p*-values < 0.05).

### Performance comparison

**Figure 2:**
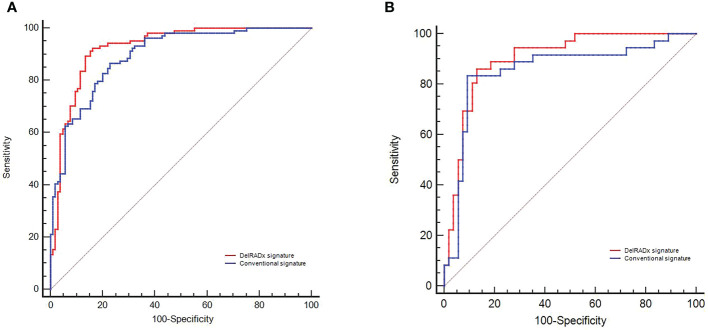
The predictive performance of the DelRADx signature and conventional radiomics signature for each patient in the training set **(A)** and validation set **(B)**.

The DelRADx model exhibited good performance with an AUC of 0.925 (95% CI, 0.888–0.962) in the training set and 0.902 (95% CI, 0.838–0.966) in the validation set, respectively. For the conventional radiomics models, the AUC was 0.894 (95% CI, 0.853~0.936) in the training set and 0.856 (95% CI, 0.767–0.945) in the validation set, respectively. Compared with the conventional radiomics model, the DelRADx model showed significantly higher AUC in the validation set (*p* < 0.05 of the DeLong test) (**Figure 2**).

### Radiomics nomogram building and evaluation

**Figure 3:**
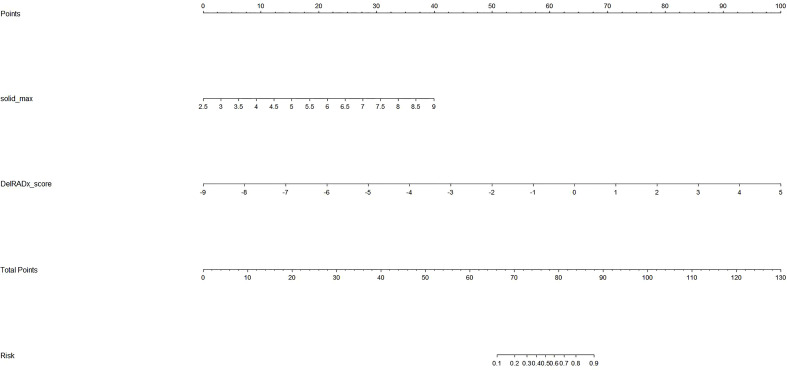
The integrated nomogram incorporating the DelRADx signature and solid_max.

To provide an easy-to-use tool, a delta-radiomics-based integrated nomogram incorporating the DelRADx signature and the solid_max was constructed using the multivariable logistical regression analysis (as shown in **Figure 3**). According to the univariate logistic regression analysis, both the DelRADx signature and the solid_max could independently predict the invasiveness of PSNs (**Table 2**). Compared with the DelRADx signature, the total IDI was significant at 0.0769 for the integrated nomogram (95% CI, 0.0394–0.1144, *p* < 0.001).

**Table 2:** Multivariate analysis of radiomics-based integrated nomogram for differentiating invasive adenocarcinoma.

Variables	Odds ratio	95% CI	*p*
Solid_max	1.23	1.82-6.39	0.0001
DelRADx signature	1.51	2.28-8.93	0.0001

**Figure 4:**
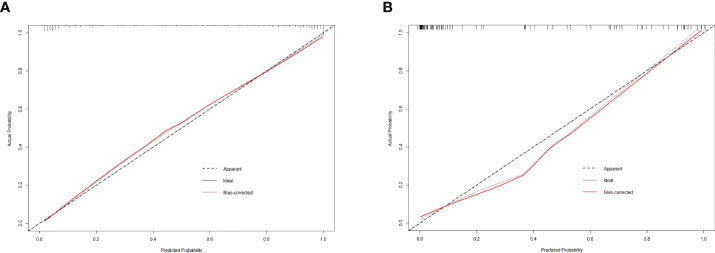
The calibration curve of the integrated nomogram in the training dataset **(A)** and validation dataset **(B)**.

**Figure 5:**
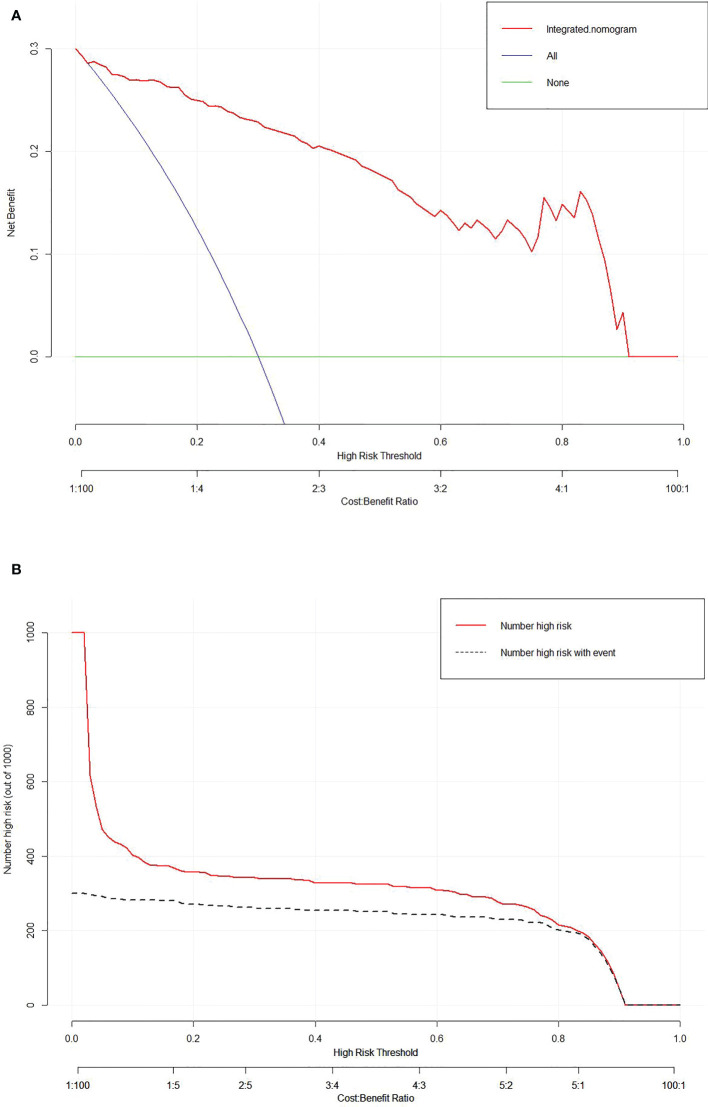
Decision curve analysis **(A)** and clinical impact plot **(B)** for the integrated nomogram in the validation set. The *x*-axis indicates threshold probability, and the *y*-axis indicates the net benefit. The red line represents the integrated nomogram.

The calibration curve of the integrated nomogram demonstrated favorable agreement with actual observation in the training cohort and was confirmed in the validation set with a mean absolute error of 0.001 and 0.019, respectively (**Figure 4**). Decision curve analysis and clinical impact plot indicated that if the threshold probability of a patient was within 90%, using the integrated nomogram to predict IAC added more benefit than either the treat-all-patient scheme or the treat-none scheme (**Figures 5A, B**).

## Discussion

In this study, we developed and validated a diagnostic DelRADx signature in the individualized evaluation of invasive adenocarcinomas for patients with PSNs. Our results demonstrated that the proposed DelRADx model outperformed the conventional radiomics model based on NCECT with an AUC of 0.925 in the training set and 0.902 in the validation set, respectively. Furthermore, our easy-to-use nomogram incorporating the DelRADx signature and radiographic independent predictor facilitated the performance compared with the DelRADx model alone. Our findings indicate that the DelRADx signature could provide additional information for invasive adenocarcinomas and, consequently, help to provide better support for decision-making when treating patients with PSNs.

The association between the solid component and pathological invasion has been extensively researched (12, 13). Several studies suggest good prediction of IACs if the threshold of the solid component is >5 mm (14). Our data are in keeping with previous observations that the diameter of solid components is a significant independent differentiator for IACs. While in our investigation, the prediction threshold was preshifted to 4.5 mm. The observed discrepancy could be explained in part by the fact that the current study included not only the subcohort of MIA but also a sample of AIS, which contained fewer solid components. Similarly, Luo et al. (15) developed a model with pleural indentation, solid component size, and solid component proportion for differentiating IAC from non-IAC in patients with PSNs and achieved an AUC of 0.85. Weng et al. (16) used lesion shape and solid component size to create a prediction model for PSNs with an AUC of 0.76.

Despite the predictive power of the radiographic features, the accurate identification of IACs with subjective assessment remains challenging in clinical practice. Radiomics is a high-throughput and automatic analysis technique for medical images. Numerous studies have proved that the development of radiomics represents a significant breakthrough in overcoming the limitation of subjective evaluation (17). Delta radiomics have shown potential advantages in earlier diagnosis and prognosis estimation in a variety of tumors.

In this study, we constructed an individualized delta-radiomics model to predict the invasiveness of PSNs. The results indicated that our delta-radiomics signature derived from CECT and NCECT outperformed the conventional radiomics signature. One of the potential explanations is that the images obtained from CECT could reveal the density of microvessels, which is closely linked with tumor invasion (18). Additionally, delta-radiomics could provide supplementary information on intratumoral heterogeneity, which is also supporting evidence for tumor invasion.

Previous studies support our interpretation. Son et al. (19) found that CECT imaging metrics could add value in distinguishing invasive adenocarcinoma from AIS/MIA that manifested as PSNs. For the DelRADx analysis, Wang et al. investigated a delta-radiomics model derived from the positioning CT and resetting CT after radiation therapy, and the result demonstrated that the delta-radiomics signature could be a promising image biomarker for the prediction of severe acute radiation pneumonitis. In line with our study, Saeed et al. (11) indicated that the Rider features in the machine learning-based delta-radiomics model could improve the performance of lung cancer screening. Our study found that the delta-radiomic features of the gray-level run-length matrix (GLRLM) had the added value in differentiating the invasiveness of lung adenocarcinoma. As is well-known, GLRLM is used to describe the distribution of texture changes between neighboring pixels. In our research, the change of GLRLM for IACs was significantly higher than for AIS/MIA, which verified the higher heterogeneity or asymmetry for IACs.

We further developed a novel delta radiomics-based nomogram by integrating the DelRADx signature with a radiographic independent predictor for convenient access in clinical applications. Our proposed nomogram showed facilitated performance with an IDI of 0.077 compared with the DelRADx model alone. Meanwhile, the developed tool revealed favorable calibration performance in the calibration curve analysis. Additionally, the decision curve analysis and clinical impact plot illustrated the potential clinical application value of our nomogram. Our findings concurred with the observations that delta radiomics incorporated with clinical data could improve the performance of the prediction model. Khorrami et al. (20) incorporated DelRADx and perinodular morphological characteristics into an individualized decision-making model for patients with advanced NSCLC, and the results showed that the noninvasive approach could effectively identify the candidates who benefit from immunotherapy. Notably, the data used in our model was all clinically accessible, requiring no more investigations and expenditures.

There are several limitations to this study. Firstly, inherent biases are inevitable in this retrospective, single-center analysis. For instance, the PSNs that were examined without CECT were excluded from this study. Secondly, although manual segmentation is regarded as the golden criterion for ROI, it is a labor- and time-intensive method that limits its application to a larger cohort. Thirdly, the reproducibility of radiomic features may face additional challenges due to the variance from different scanner parameters. Last but not the least, the stability of radiomic features may be affected by different acquisition phases of CECT scanning. A multicenter study with a prospective design is planned for our future study.

In conclusion, the delta-radiomics signatures can significantly improve the performance in the differential diagnosis of IACs from AIS/MIAs in patients with PSNs. The delta-radiomics-based nomogram coupled with radiographic features may serve as a convenient way in providing highly informative data for clinical decision support.

## Data availability statement

The original contributions presented in the study are included in the article/**Supplementary Material**. Further inquiries can be directed to the corresponding authors.

## Ethics statement

This study design was approved by the institutional research ethics board of Huadong hospital, and the informed consent requirement was waived for the retrospective research with anonymous data.

## Author contributions

Data curation: DBM, HW. Formal analysis: WFC, ZXM. Funding acquisition: ML. Methodology: RZW. Software: WFC, ZXM. Supervision: YQH. Validation: CL. Visualization: YLY. Writing - original draft: WFC. Writing - review and editing: ML. All authors contributed to the article and approved the submitted version.

## Funding

This study was funded by the National Natural Science Foundation of China (grant no.: 61976238) and Research Project Plan of Shanghai Municipal Health Commission (grant no.: 20214Y0309).

## Conflict of interest

The authors declare that the research was conducted in the absence of any commercial or financial relationships that could be construed as a potential conflict of interest.

## Publisher’s note

All claims expressed in this article are solely those of the authors and do not necessarily represent those of their affiliated organizations, or those of the publisher, the editors and the reviewers. Any product that may be evaluated in this article, or claim that may be made by its manufacturer, is not guaranteed or endorsed by the publisher.
