# Supplementary material for: A delta-radiomics model for preoperative prediction of invasive lung adenocarcinomas manifesting as radiological part-solid nodules
Source: Front Oncol. 2022 Nov 16;12:927974. doi: 10.3389/fonc.2022.927974 (PMC9709341; doi:10.3389/fonc.2022.927974)
Supplement: Supplementary file 1 [file DataSheet_1.docx]

**Table S1. scanning parameters**

| parameters |  | GE Discovery CT750 HD |  | Somatom Definition Flash |
| --- | --- | --- | --- | --- |
| Tube voltage（kVp） |  | 120 |  | 120 |
| Tube current(mAs) |  | 200 |  | 110 |
| Pitch |  | 0.984:1 |  | 1.0 |
| Collimation(mm) |  | 0.625*64 |  | 0.6*64 |
| Rotation time(s/rot) |  | 0.5 |  | 0.33 |
| SFOV(cm) |  | 50 |  | 50 |
| Slice thickness of reconstruction(mm) |  | 1.25 |  | 1 |
| Slice interval of reconstruction(mm) |  | 1.25 |  | 1 |
| Reconstruction algorithm |  | STND |  | Medium sharp |

Surgically resected participants with SPNs [retrospective](javascript:;)ly reviewed from [January](javascript:;) 2017 to [December](javascript:;) 2021(n= 2171)

Participants with histological results other than lung adenocarcinoma([benign](javascript:;)or [squamous carcinoma](javascript:;),et al) (n=453)

Participants with histological results of lung adenocarcinoma (n=1718)

SPNs manifested as solid and pGGNs(n= 1154)

SPNs manifested as PSNs(n= 566)

Participants with PSNs

obtained NCECT examination only(n= 224)

Participants with PSNs obtained both NCECT and CECT examinations

(n= 342)

Excluded (n= 43)

1)nodule size smaller than 6mm (n=22)

2)Poor image quality(n= 8)

3)fail to extract the radiomics features for the unknown reasons (n= 8)

4)multiple PSNs without the pathological conclusive result (n= 5)

Total included (n= 299)

**Fig. S1.** **the flowchart of inclusion and exclusion**

In total, 740 DelRADx were [calculat](javascript:;)ed based on the NCECT and CECT radiomics features.

After removing 14 strongly correlated features, 316 DelRADx were

selected for the follow-up analysis(Spearman Rho＜0.90).

In the univariate analysis, 330 DelRADx were statistically significantly associated with IAC(P < 0.05).

A total of 699 features with ICCs > 0.8 were reserved according to the re-segmentation data( ICC:0.8031-0.9998).

ICC

ANOVA/MW

Spearman rank correlation analysis

LASSO-logistic

Nine robust DelRADx were ultimately selected.

**Fig. S2. the flowchart of DelRADx selection**

**DelRADx signature**

==1.42

-4.642*original_shape_SurfaceVolumeRatio

+5.467*square_gldm_SmallDependenceHighGrayLevelEmphasis

-5.84*squareroot_gldm_LargeDependenceEmphasis

-2.798*wavelet-HHH_firstorder_Kurtosis

+3.945*wavelet-HHL_glcm_MCC

-6.396*wavelet-HHL_glrlm_HighGrayLevelRunEmphasis

+4.551*wavelet-HHL_glszm_ZoneVariance

-2.983*wavelet-HLH_firstorder_Skewness

-3.588*wavelet-LLH_glszm_HighGrayLevelZoneEmphasis

**Conventional radiomics signature**

=5.63

+3.619*exponential_glrlm_ShortRunLowGrayLevelEmphasis

-8.076*log-sigma-3-0-mm-3D_ngtdm_Coarseness

-7.935*square_glcm_SumAverage

-4.976*wavelet-HHH_firstorder_Skewness

-5.454*wavelet-LHL_glrlm_ShortRunEmphasis

**Fig. S3. the signature formulas of DelRADx and conventional radiomics**

| **Table S2.** Multivariate analysis of radiographic model for differentiating invasive adenocarcinoma | | | | |
| --- | --- | --- | --- | --- |
| Variables |  | Odds Ratio |  | P |
| solid_max |  | 3.37 |  | 0.002 |
| nodule_max |  | -0.63 |  | 0.076 |
| CTR |  | -11.69 |  | 0.223 |
